# Interleukin-6 is associated with acute concussion in military combat personnel

**DOI:** 10.1186/s12883-020-01760-x

**Published:** 2020-05-25

**Authors:** Katie A. Edwards, Jessica M. Gill, Cassandra L. Pattinson, Chen Lai, Misha Brière, Nicholas J. Rogers, Denise Milhorn, Jonathan Elliot, Walter Carr

**Affiliations:** 1 National Institute of Nursing Research, National Institutes of Health, 3 Center Drive, Building 3, Room 26E, Bethesda, MD 20892 USA; 2 Henry M. Jackson Foundation for the Advancement of Military Medicine, 6720A Rockledge Dr, Bethesda, MD 20817 USA; 3 CNRM Co-Director Biomarkers Core, Uniformed Services University of the Health Sciences, Bethesda, USA; 4 87th Medical Group, Joint Base McGuire-Dix-Lakehurst, 3458 Neely Road, Trenton, NJ 08641 USA; 5 United States Army Research Institute of Environmental Medicine, 10 General Greene Ave, Natick, MA 01760 USA; 6 USS Gerald R. Ford (CVN78), FPO, AE, Norfolk, VA 09523 USA; 7 Oak Ridge Institute for Science and Education, Oak Ridge, TN USA; 8 Walter Reed Army Institute of Research, 503 Robert Grant Avenue, Silver Spring, MD 20910 USA

**Keywords:** Inflammatory cytokines, Concussion, Biomarkers

## Abstract

**Background:**

Concussion is the most common type of TBI, yet reliable objective measures related to these injuries and associated recovery processes remain elusive, especially in military personnel. The purpose of this study was to characterize the relationship between cytokines and recovery from acute brain injury in active duty service members. Inflammatory cytokines (IL-6, IL-10, and TNFα) were measured acutely in blood samples within 8 h following a medically diagnosed concussion and then 24 h later.

**Methods:**

Participants (*n* = 94) were categorized into two groups: 1) military personnel who sustained provider-diagnosed concussion, without other major medical diagnosis (*n* = 45) and 2) healthy control participants in the same deployment environment who did not sustain concussion or other illness or injuries (*n* = 49). IL-6, IL-10, and TNFα concentrations were measured using an ultrasensitive single-molecule enzyme-linked immunosorbent assay. Differences in cytokine levels between concussed and healthy groups were evaluated at two time points (time point 1 ≤ 8 h after injury; time point 2 = 24 h following time point 1).

**Results:**

At time point 1, IL-6 median (IQR) concentrations were 2.62 (3.62) in the concussed group, which was greater compared to IL-6 in the healthy control group (1.03 (0.90); U = 420.00, z = − 5.12, *p* < 0.001). Compared to healthy controls, the concussed group did not differ at time point 1 in IL-10 or TNFα concentrations (*p*’s > 0.05). At time point 2, no differences were detected between concussed and healthy controls for IL-6, IL-10, or TNFα (*p*’s > 0.05). The median difference between time points 1 and 2 were compared between the concussed and healthy control groups for IL-6, IL-10, and TNFα. Change in IL-6 across time was greater for the concussed group than healthy control (− 1.54 (3.12); U = 315.00, z = − 5.96, *p* < 0.001), with no differences between groups in the change of IL-10 or TNFα (*p*’s > 0.05).

**Conclusion:**

Reported here is a significant elevation of IL-6 levels in concussed military personnel less than 8 h following injury. Future studies may examine acute and chronic neurological symptomology associated with inflammatory cytokine levels, distinguish individuals at high risk for developing neurological complications, and identify underlying biological pathways to mitigate inflammation and improve outcomes.

## Background

Concussion, or mild traumatic brain injury (mTBI), is the most common type of TBI across all age ranges, yet reliable biomarkers related to these injuries and associated recovery processes remain elusive, especially in military personnel [1, 2]. At this time, limited objective measures exist for identification of individuals who may be at high risk for developing complications and poor outcomes following concussion. Improving our understanding of underlying biological changes that occur following a concussion or mTBI is crucial to identify people who may be at the most risk of poor outcomes, who require increased monitoring and preventive interventions, and for ongoing monitoring of these individuals [3].

For the military, identifying blood-based markers for concussion has been a 20-year program of research. Concussion is recognized as one of the most prevalent injuries among military members who served in Operation Iraqi Freedom [4] and Operation Enduring Freedom (OEF). A key factor in the rate of concussion among military casualties is adversaries’ increasing use of improvised explosive devices (IEDs) [5]. Blast exposure affects multiple organs and tissues, including the central nervous system, which is well documented in preclinical models [6]. Over time, blast exposures as well as blunt force injuries are associated with neurological symptoms that are garnering concern for the health and well-being of military personnel and veterans [7–9].

Blood-based biomarkers show promise in distinguishing patients with traumatic brain injuries who require additional monitoring and interventions, as evidenced by the recent FDA-approval for blood biomarkers ubiquitin C-terminal hydrolase (UCH-L1) and glial fibrillary acidic protein (GFAP) to inform clinical CT decisions [10]. Additional investigation of blood biomarkers related to secondary pathological processes initiated by TBIs may elucidate pathological pathways for research and eventual clinical applications [11, 12]. Specifically, studies of blood-based inflammatory protein biomarkers implicates underlying inflammatory processes following TBIs of all severities that are important for acute recovery [13, 14] and may relate to chronic symptoms [15, 16]. Not only may inflammatory biomarkers help monitor and predict outcomes, but they also identify inflammatory pathways that could be targeted for therapies [17]. Of key interest are pro- and anti-inflammatory cytokines, as they have been implicated in the underlying balance of inflammatory processes which occur following a TBI [11, 18]. For example, preclinical brain injury studies of interleukin (IL)-6 indicate some IL-6 activity is beneficial for recruiting immune cells and improving outcomes, especially in the acute phase [19]. However, harmful outcomes may result from either IL-6 deficiency, as demonstrated in IL-6 knockout mice [20], or chronic IL-6 overexpression [17, 19]. Likewise, study of IL-10 in preclinical brain injury models has shown poor outcomes in IL-10 knockout mice, with IL-10 administration improving neurological function and decreasing lesion volume [21]. In human studies, inflammatory markers, including levels of IL-6, IL-10, and TNFα, are observed to be elevated and associate with poor outcomes in moderate and severe cases of TBI [13, 14, 16, 22–29].

Evaluation of cytokine profiles following concussions are increasing in recent years [30–33]. Previously reported are elevated levels of plasma inflammatory cytokines, IL-6 and TNFα, concurrent with chronic neurological symptomology among military personnel who experienced blunt force and/or blast injury (within 16 months of deployment) [31]. This lab also reported an association between blast exposure and acute increases in levels of IL-6 and TNFα in an undiagnosed military blast training population [34]. These studies suggest that mild injuries have similar inflammatory changes to preclinical and severe human TBI studies. Concussion is the most common type of TBI and a recognized threat for military personnel deployed to a combat zone; yet, blood levels of IL-6, IL-10, and TNFα have not been studied during the first 24 h following concussion sustained during a military combat deployment. The previous literature implicates that appropriate inflammatory response is important to successful outcomes; and over or under expression of the cytokine response may contribute to poor outcomes. Thus, protein blood biomarkers, including cytokines, may be useful in monitoring for poor outcomes, which may be especially beneficial among concussed individuals who may not otherwise follow up on mild subjective symptoms [35].

To better understand the role of inflammatory cytokines in concussions and implications for service member health, cytokines levels of IL-6, IL-10, and TNFα were measured acutely in blood samples within 8 h following a medically diagnosed concussion and then again 24 h later to characterize the relationship between cytokines and recovery from acute brain injury. Findings from this line of research will provide the basis to identify the biological underpinnings of inflammatory processes occurring in the acute stage of recovery from concussion sustained in austere environments like military deployment, which is necessary to improve medical decision making and recovery trajectories.

## Methods

### Participants

This study protocol was reviewed and approved by the Research Institutional Review Boards at the US Army Medical Research and Development Command and the Walter Reed Army Institute of Research. Each study participant provided written informed consent for participation. This unique, observational cohort study consisted of: 1) deployed military personnel who sustained a concussion, provider diagnosed, without other major medical diagnosis and received acute medical care (*n* = 45) and 2) healthy control participants in the same deployment environment who did not sustain concussion or other illness or injuries (*n* = 49). Participants were referred to this protocol by treating providers as having concussion and no other major injury. Exclusion criteria for the control group included self-report of illness, pain, or history of head injury within previous 4 weeks. Both groups were deployed to units in the same region of operations in Afghanistan. The environment and process for diagnosis was comparable to a standard civilian emergency room. Concussion was defined as the reporting of an injury event, a normal structural neuroimaging by head CT or conventional brain MRI, and at least one of the following: alteration of consciousness less than 24 h; loss of consciousness, if any, for less than 30 min; or post-traumatic amnesia for less than 24 h. Providers making diagnoses all held MD or DO degree and were working according to Department of Defense protocol [36].

Participants had blood draws at two time points: 1) time point 1 was at the time of medical care, less than 8 h after concussion, or at the time of initial encounter for the healthy control group and 2) time point 2 was 24 h following the time of the first blood draw. There was some attrition in each of the two cohorts between time point 1 and time point 2. For this analysis, we included only participants with samples at both time points.

### Blood sampling

Whole blood was drawn and processed for serum from April–November 2012 in Southwest Afghanistan, using standard protocols, within 1 hour of the blood draw. Serum was aliquoted and frozen on site at − 80 °C. Frozen samples were shipped to the Walter Reed Army Institute of Research (WRAIR) in Maryland, USA at 4 separate time points across the collection period. Shipping was made with dry ice and a container designed for this purpose. Each shipment included a thermometer that continuously logged temperature during the shipment period. Upon receipt at WRAIR, shipments were transferred to continuously powered − 80 freezers that are maintained according to WRAIR protocols. The final transfer of samples was approximately 5 miles from WRAIR to the National Institutes of Health, National Institute of Nursing Research (NINR) via insulated container and dry ice packing. Upon receipt at NINR, samples were transferred to NINR freezers and maintained according to protocol until thawed for assay from July to October 2017.

### Laboratory methods

IL-6, IL-10, and TNFα concentrations were measured using Simoa™ technology (Quanterix, Lexington, MA), an ultrasensitive single-molecule enzyme-linked immunosorbent assay, as previously described [37]. The IL-6, IL-10, and TNFα assays have low limits of detections (0.006 pg/mL, 0.0022 pg/mL, and 0.011 pg/mL, respectively). Samples were run in duplicate, and the personnel running analyses were blinded to group. Average coefficients of variation (CV) were 4.75, 4.43, and 4.78% for IL-6, IL-10 and TNFα, respectively. Four samples with CV > 15% were excluded from analysis, and are not included in the cohort sizes.

### Statistical methods

SPSS version 25 (IBM Corporation, Chicago, IL) was used to conduct statistical analyses, and GraphPad Prism version 7.0d (Graph Pad Software, San Diego, CA) was used to create figures. Baseline demographic characteristics were compared between healthy and concussed groups using Pearson’s chi square (race and gender) and ANOVA (age). Distributions did not require adjustment for normality. The differences in concentrations of IL-6, IL-10, and TNFα between the concussed and healthy groups at the first time point and at the second time point (time point 1 = < 8 h after injury; time point 2 = 24 h following time point 1) were compared using Mann Whitney U tests. For the difference between the two time points, mean difference was calculated by subtracting each participant’s cytokine concentration at time point 2 from the concentration level at time point 1, resulting in a variable that reflects the total change in each cytokine across 24 h. A Mann Whitney U test was conducted to evaluate if there was a significant difference in time-based change for the cytokines between the concussed group and the healthy control group. Because the two groups were similar in demographic characteristics, we did not include race or age covariates in these models.

## Results

### Demographics

All participants were active duty service members (*n* = 94) deployed to Afghanistan, and the sample was primarily male (96.8%). The mean age was 26.41 years (SD = 6.364) with a range of 19 to 48 years of age. Military personnel who were medically diagnosed with a concussion and received acute care (*n* = 45) were compared to healthy controls with no diagnosis of concussion (*n* = 49) deployed to the same combat station. The two groups did not differ in demographic features including sex, race, or age (see Table 1). All participants within both groups had a Glasgow Coma Score (GCS) of 15 at the time of study enrollment. The concussed personnel were evaluated and diagnosed by a healthcare provider according to standard of care and were then referred to study personnel < 8 h following injury. Of the concussed personnel, 33 (73.3%) participants were exposed to blast during the injury event, and the others reported a blunt force injury without exposure to blast (see Table 2).

**Table 1:** Demographic Data

	Healthy Controls (*N* = 49)	Concussion (*N* = 45)	Significance
Age in years, M *(SD)*	26.63 (6.978)	26.36 (5.747)	*p* = 0.841^a^ F = 0.041^a^
Sex, No. (%)			*p* = 0.066^b^
Male	49 (100)	42 (93.3)	
Female	0 (0)	3 (6.7)	
Race, No. (%)			*p* = 0.297^b^
White	35 (71.4)	21 (67.7)	
Black	5 (10.2)	0 (0.0)	
Hispanic	6 (12.2)	5 (7.5)	
Pacific Islander	2 (4.1)	1 (3.2)	
Asian	1 (2.0)	1 (3.2)	
Middle Eastern	0 (0.0)	1 (3.2)	
Other	0 (0.0)	2 (6.5)	

*Note.* The percentages in each column refer to the proportion of individuals in each sex and race category. Race for 14 participants in the concussed group is missing information in the data. ^a^Anova ^b^Pearson’s chi square *p value significant at the *p* < 0.05 level

**Table 2:** Clinical Data

Reason for visit	Concussion (*N* = 45)
Blast exposure, No. (%)	33 (73.3)
Blunt force injury, without blast, No. (%)	12 (26.7)

*Note.* The percentages in each column refer to the proportion of individuals with each reason for visit

### Inflammatory protein differences following concussion

#### Between-group comparisons at time point 1 and at time point 2

**Fig. 1:**
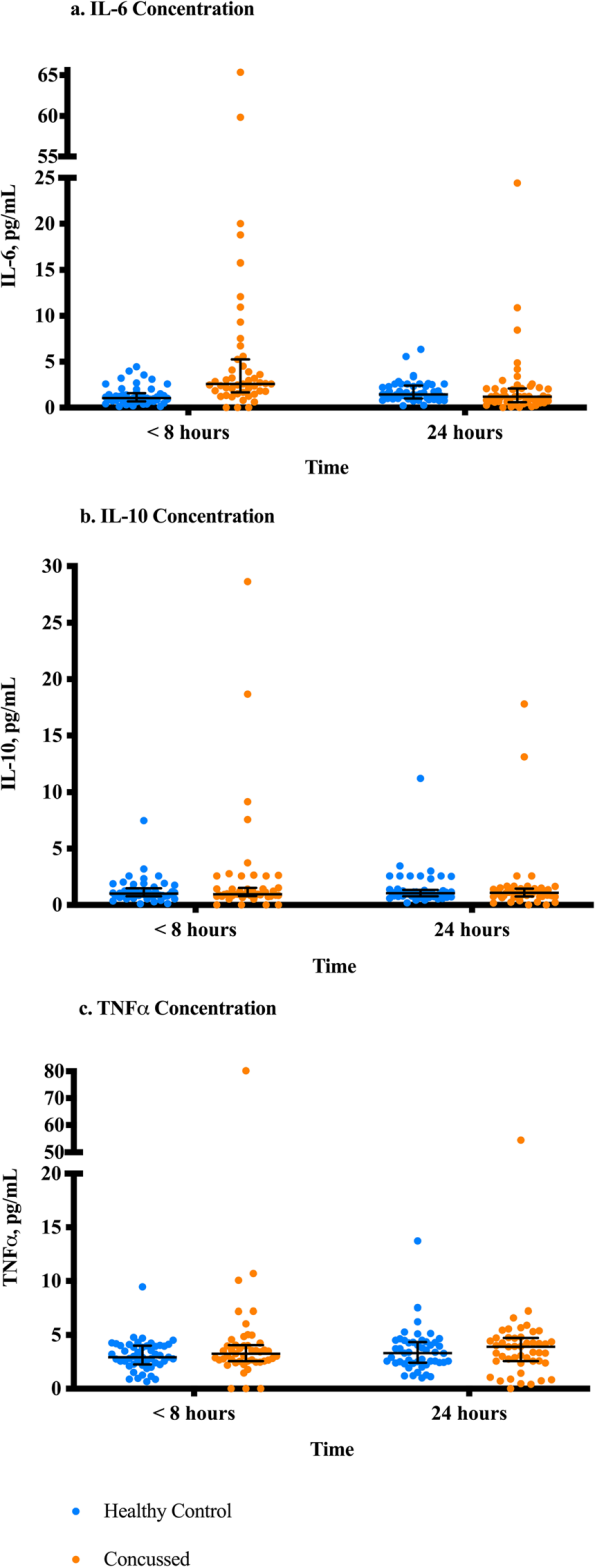
**a**-**c**. Between-Group Comparisons of Cytokines between Concussed and Healthy Controls at Two Time Points. Note. Time point 1 is <8 h after injury. Time point 2 is 24 h after time point 1. Mann Whitney U tests were conducted to compare each cytokine’s concentration between the healthy and concussed groups at each time point for **a**. IL-6, **b**. IL-10, and **c**. TNFα. IL-6 concentration was significantly higher in the concussed group at time point 1 at *p*<0.001

**Fig. 2:**
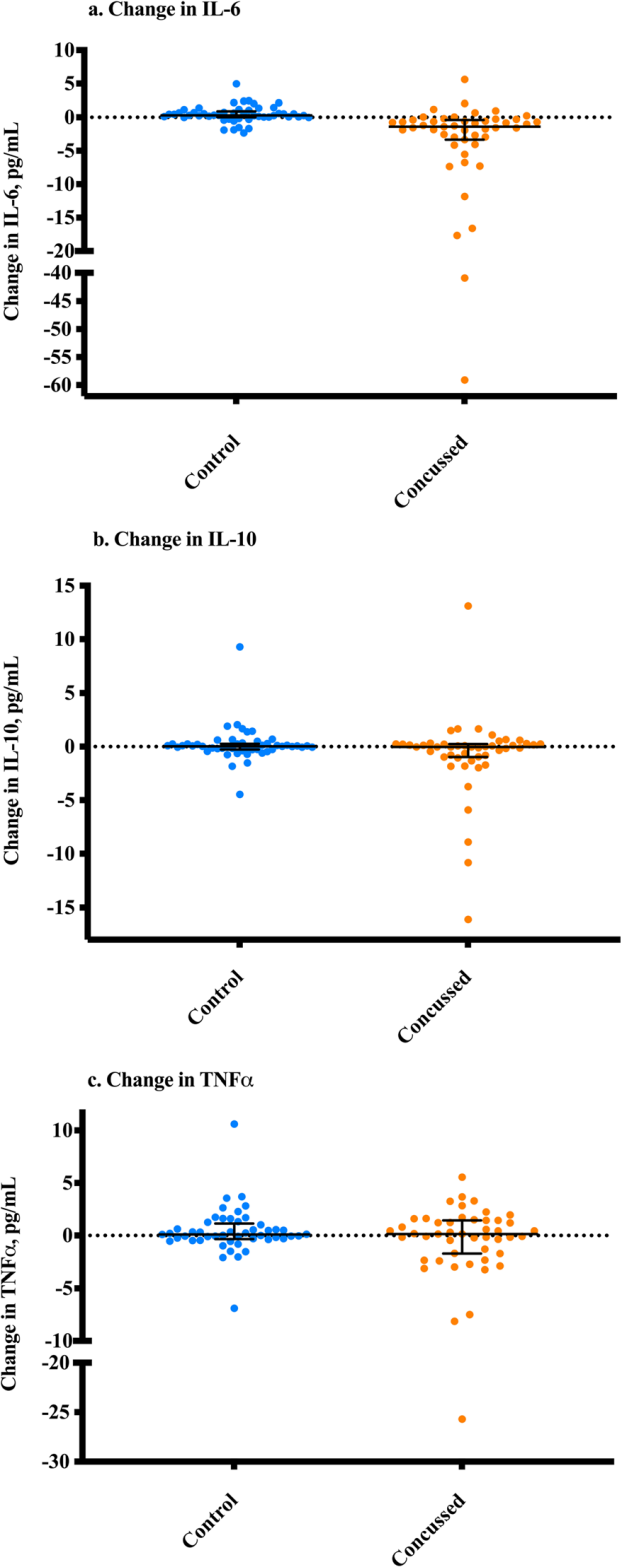
**a**-**c**. Median Difference in Each Cytokine Over Time from Time Point 1 to Time Point 2. Note. Time point 1 is <8 h after injury. Time point 2 is 24 h after time point 1. Mean difference = (each participant’s cytokine concentration at time point 2) - (each participant’s concentration level at time point 1). Mann Whitney U tests were conducted to determine if there were differences in the mean change variable between these groups. **a**. IL-6, **b**. IL-10, and **c**. TNFα. IL-6 was significantly different in the concussed group as compared to the healthy control at *p*<0.001

Differences in IL-6, IL-10, and TNFα between concussed and healthy groups were evaluated at each time point (time point 1 = < 8 h after injury; time point 2 = 24 h following time point 1). At time point 1, IL-6 median (interquartile range, IQR) concentrations were 2.62 (3.62) in the concussed group, which was greater compared to the IL-6 concentrations of 1.03 (0.90) in the healthy control group (*U* = 420.00, z = − 5.12, *p* < 0.001) (see Fig. 1a). The two highest levels of IL-6 were samples collected > 4 h after injury. Compared to healthy controls, the concussed group did not differ at time point 1 in concentrations of IL-10 (*p* = 0.358) or TNFα (*p* = 0.382) (see Fig. 1b-c). At time point 2, no differences were detected between concussed and healthy controls for IL-6 (*p* = 0.075), IL-10 (*p* = 0.937), or TNFα (*p* = 0.390) concentrations (see Fig. 1a-c). The median difference between time point 1 and time point 2 was compared between the concussed and healthy control groups for IL-6, IL-10, and TNFα. A Mann Whitney U test showed that the change in IL-6 across time was greater for the concussed group than the healthy control (− 1.54 (3.12); *U* = 315.00, z = − 5.96, *p* < 0.001) (see Fig. 2a). There was no difference between groups in the change of IL-10 (*p* = 0.158) or TNFα (*p* = 0.777) (See Fig. 2b-c). The percentage change in IL-6 was − 67.7% in the concussed group compared to 33.5% in the healthy controls.

#### Within-group comparisons at time point 1 and time point 2

**Fig. 3:**
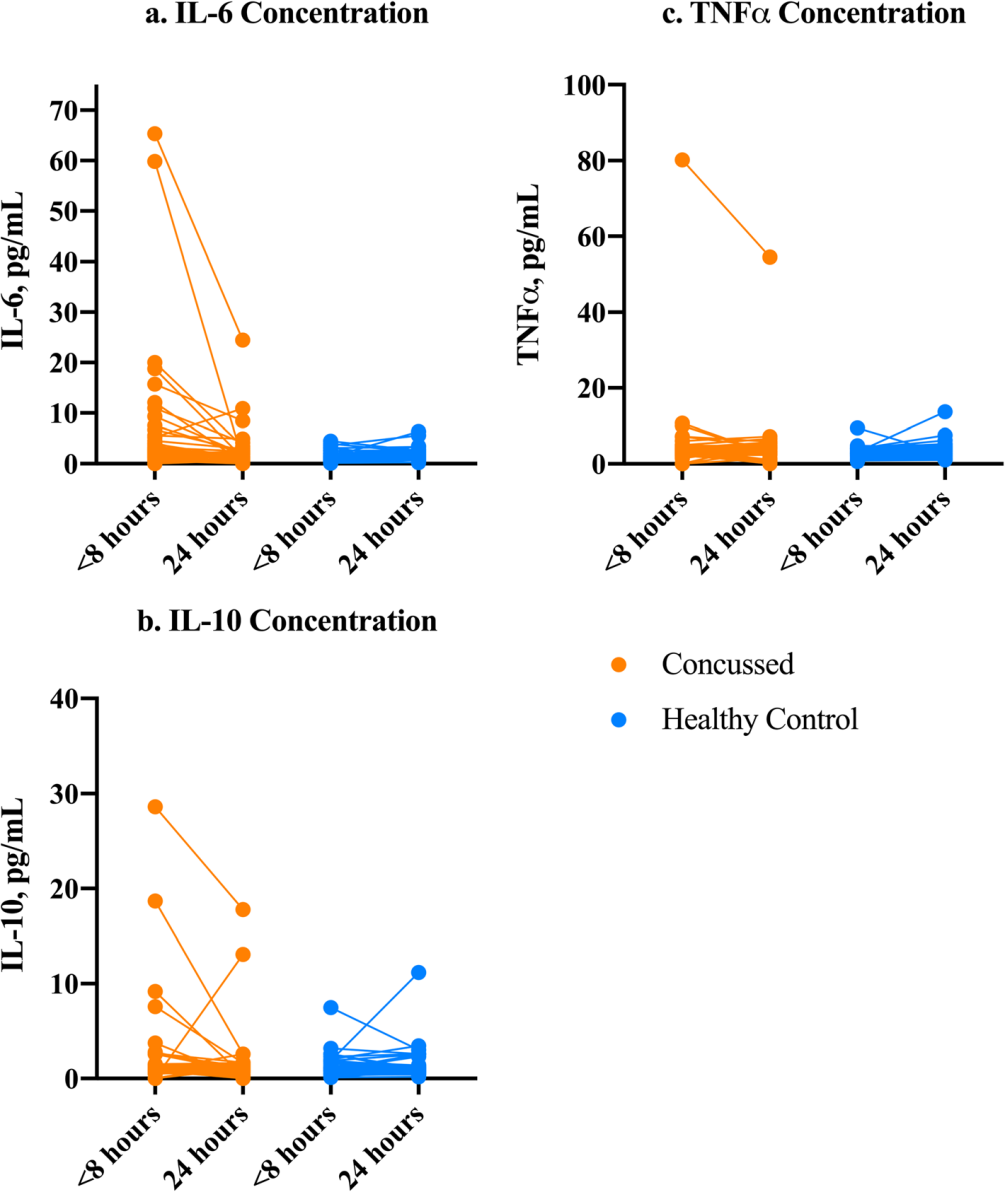
**a**-**c**. Within-Group Comparisons of Cytokines between Concussed and Healthy Controls at Two Time Points. Note. Time point 1 is <8 h after injury. Time point 2 is 24 h after time point 1. Within-groups differences were calculated used Wilcoxon Sign-Rank Tests comparing each cytokine’s concentration between the healthy and concussed groups at each time point for **a**. IL-6, **b**. IL-10, and **c**. TNFα. IL-6 concentration was significantly higher in the concussed group at time point 1 as compared to time point 2 (z = -4.76, *p*<0.001)

Within-group differences in IL-6, IL-10, and TNFα between concussed and healthy groups were evaluated at each time point using the Wilcoxon Sign-Rank Test. Change in IL-6 between time points 1 and 2 in the concussed group remains statistically significant (z = − 4.76, *p* < 0.001) (Fig. 3a). In the healthy control group, there was a significant increase in IL-6 from time point 1 to time point 2 (z = − 3.17, *p* = 0.002). However, the change was in the opposite direction for the concussed group and the effect size for the concussion group was larger (0.5) than for the healthy control group (0.3). There were no differences in the concussed group for IL-10 (z = − 1.41, *p* = 0.16) or TNFα (z = − 0.31, *p* = 0.76), and there were no differences in the healthy control group for IL-10 (z = − 0.58, *p* = 0.56) or TNFα (z = − 1.46, *p* = 0.145) (Fig. 3b-c). 13.3% (6 of the 45 individuals with concussion) of the concussed sample values at time point 1 were within the IQR of the healthy control group (0.68–1.58), and 80% (36 of the 45 individuals with concussion) of the concussed group was above the IQR of the healthy control group at time point 1 (> 1.58).

#### Area under the curve

**Fig. 4:**
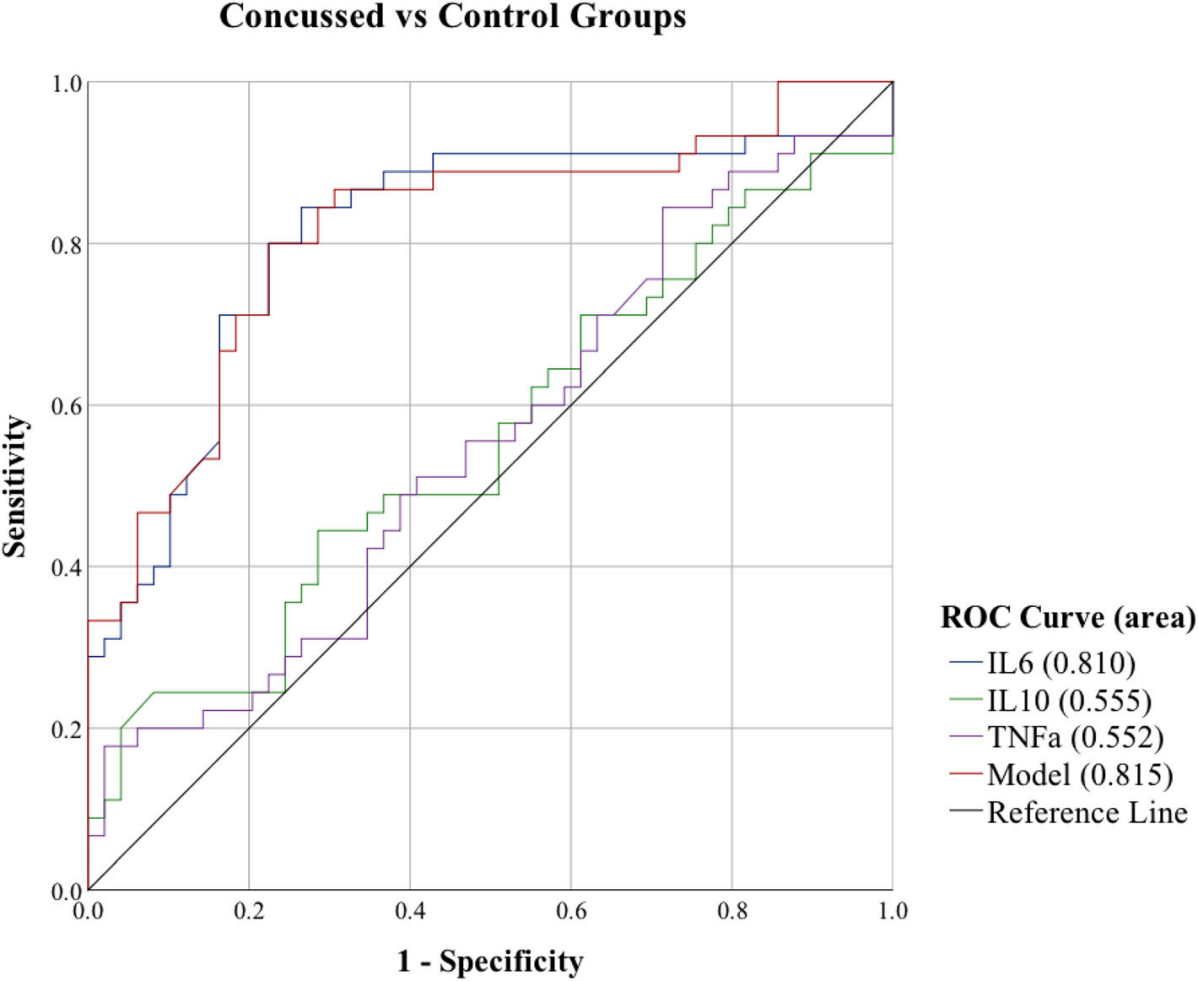
Sensitivity of Acute Cytokines to Predict Concussed vs Control Groups. *Note.* Receiver operating characteristic (ROC) curves at time point 1 for IL-6, IL-10, TNFα, and the combined model which includes all biomarkers

Binomial logistic regression provided individual IL-6, IL-10, TNFα, and combined biomarker model ROC curves. To determine the ability of the cytokines to differentiate concussed and healthy control groupings at time point 1, an area under the curve (AUC) analysis was performed (Fig. 4). IL-6 was a significant predictor and had a good AUC value (AUC 0.81, 95% CI 0.72–0.90), and the combined biomarker model showed good discriminatory power (AUC 0.82, 95% CI 0.73–0.90). IL-10 (AUC 0.56, 95% CI 0.44–0.67) and TNFα (AUC 0.55, 95% CI 0.44–0.67) were not significant.

## Discussion

The finding of higher IL-6 within 8 h of a medically diagnosed concussion sustained during combat deployment is consistent with previous studies that report acutely elevated levels of IL-6 in severe TBI patients [13, 14, 22]. In fact, this study is the first one known that reports acute biomarker findings in a deployed cohort of military personnel with concussion. There are a variety of factors that make a deployed population unique, and for this study, paramount is the high rate of blast exposure. This finding is in line with this lab’s previous report that linked IL-6 elevations to a blast exposure sustained during training by an undiagnosed population which did not include blunt force. That elevation was then followed by a decrease in IL-6 in sampling on subsequent days to below baseline levels [34]. Therefore, the present findings indicate that concussions sustained during deployment, highly comorbid with blast, result in elevations of IL-6, followed by a decrease in concentrations within 24 h. This finding implicates a role for IL-6 in recovery from concussions, as well as blast exposures, and that understanding these complex relationships may be important to improving care provided to military personnel with complex, and often overlapping injuries sustained in combat stations.

IL-6 is involved in the modulation of inflammatory activity following a TBI, with evidence pointing to the importance of the balance of pro- and anti-inflammatory levels in the promotion of recovery following TBIs and concussions [17]. Pro-inflammatory cytokines (including IL-1, IL-6, and TNFα) and anti-inflammatory cytokines (including IL-10 and IL-1 receptor agonist, IL-4) are released by a myriad of cells, including microglia, astrocytes, macrophages, and other peripheral and CNS cells, and orchestrate the acute inflammatory response to brain injury [11, 24, 38]. Anti-inflammatory cytokines shift the balance toward neuroregenerative and neuroprotective biological pathways, and pro-inflammatory cytokines shifting the balance toward apoptosis and cell death [38], and the threshold at which this balance becomes maladaptive is not fully understood [11]. Activation of microglia induces production of neuroprotective agents including IL-10 which suppresses the proinflammatory immune response [39]. In support of this inflammatory response balance, preclinical models that knock out IL-6 activity result in poor behavioral performance following a TBI [40] as well as increased apoptosis and delayed neuronal regeneration [20], implying an important pro-inflammatory function for IL-6 immediately following injury. However, detrimental outcomes do occur with microglial activation and elevated prolonged proinflammatory response [41]. IL-6 activity following TBI has been linked to delays in motor coordination and neuronal tissue repair in preclinical models [19, 42]. There is also evidence of increased IL-6 concentration in human post-mortem brain tissue obtained following a severe TBI that resulted in mortality, compared to patients who died from non-central nervous system causes [43]. There may be long-term health consequences that result if IL-6 remains imbalanced, as findings of elevated IL-6 may be indicative of chronic neurological symptoms or deficits [31]. Findings from the current study show an IL-6 elevation within hours of a concussion, with the highest two IL-6 levels more than 4 h after injury. Levels are then similar to healthy controls at 24 h later, suggesting that IL-6 is playing a role in recovery from these mild injuries. Considering these early findings in concussion, additional studies with longer follow up are warranted to understand the role of IL-6 in recovery and links to long-term consequences.

IL-10 and TNFα were not significantly different between the concussed and healthy cohorts in the present study. This differs from a previous report of elevated levels of TNFα in military personnel following blast exposure, along with elevated IL-6 levels [34]. One explanation may be differences between the samples in the two studies. The previous report studied military personnel in a well-controlled training environment, with no reported incidences of blunt injuries and no medical diagnosis [34], while the present cohort included military personnel diagnosed with a concussion, including from blunt force injury. These reported differences in event characteristics may account for a lack of TNFα differences between the concussed and healthy military personnel. Likewise, IL-10 was not significantly different between the groups in the present study, a finding that replicated these previous findings [34]. Elevations concurrently in IL-6 and IL-10 have been observed in studies of severe TBI [13], and increases in serum IL-10 seem positively correlated with more severe TBI [23, 29]. The lack of an increase of IL-10 in the current concussed population is not surprising based on the previous literature. However, the lack of increase in IL-10 together with elevated IL-6 observed in this cohort suggests a state of pro-inflammatory activation. IL-10 is traditionally classified as exerting anti-inflammatory effects, while IL-6 is traditionally defined as pro-inflammatory characteristics [17, 38, 44], with some evidence for IL-6 anti-inflammatory characteristics [44]. IL-10 confers neuroprotective effects in animal models [45–47]. Potential long-term outcomes if the pro-inflammatory response were to remain elevated should be further explored in larger sampling and with in-depth clinical measures.

Inflammatory markers, such as cytokines, may be valuable in modeling the recovery process and predicting outcomes following concussion. Inflammatory processes may modulate secondary pathological processes after TBI [11]. In contrast to the development of diagnostic biomarkers, which would have vital roles in point-of-care tests and screening [10, 48], a robust understanding of the inflammatory pathways, including the threshold for maladaptive response, may lead to insights into immunomodulated therapies and targets. The current study is a unique sample and the first study to report acute cytokine blood biomarkers obtained from active duty military personnel who sustained concussions during a military deployment.

There are a number of factors in the current study that limit interpretations of these findings, including a relatively small sample size. Baseline blood samples before head injury were not collected for this study, and further research efforts should include baseline levels to ensure it is the head injury exposure influencing changes in blood biomarkers. Inclusion of another control group, who sustained bodily injuries but not head injuries, would also be informative in future work to isolate the influence of head injury on blood cytokine levels. Additional limitations in the scope of this study include a lack of symptom data and evaluation of long-term outcomes. Since exclusion criteria relied upon self-report, previous exposures to low level blast exposure cannot be ruled out within the control or concussed cohorts. The nature of the combat environment may limit specificity in the current study, as blast exposure and blunt injuries often occur concurrently in the same injury event. Differences between injury types may account for discrepancies with the literature, though it is outside the scope of this study to delineate effects of blast from blunt force injury causes.

## Conclusion

In conclusion, reported here is a significant elevation of IL-6 levels in concussed military personnel less than 8 h following injury. This is the first reported observation of blood levels IL-6, − 10, and TNFα in a combat environment to evaluate biomarker consequences of concussions sustained during combat deployments, which is a unique cohort given the high rates of comorbid blast exposure. The present finding of IL-6 elevation warrants further exploration of inflammatory cytokines in combat injuries involving concussion and blast, especially in future studies designed to account for the aforementioned limitations. Future studies may examine acute and chronic neurological symptomology associated with inflammatory cytokine levels, distinguish individuals at high risk for developing neurological complications, and identify underlying biological pathways to mitigate inflammation and improve outcomes. The present findings of elevated IL-6 may be further explored in larger cohorts, as well as to determine inflammatory pathways that may be targeted for therapies.

## Data Availability

The datasets analyzed during the current study available from the corresponding author on reasonable request.

## Abbreviations

CT: Computerized tomography
CV: Coefficient of variation
IED: Improvised explosive device
IL-6: Interleukin-6
IL-10: Interleukin-10
OEF: Operation Enduring Freedom
TNFα: Tumor necrosis factor alpha
mTBI: Mild traumatic brain injury
TBI: Traumatic brain injury
